# Estimation of Discriminative Feature Subset Using Community Modularity

**DOI:** 10.1038/srep25040

**Published:** 2016-04-28

**Authors:** Guodong Zhao, Sanming Liu

**Affiliations:** 1 School of Mathematics and Physics, Shanghai Dian Ji University, Shanghai 201306, P. R. China

## Abstract

Feature selection (FS) is an important preprocessing step in machine learning and data mining. In this paper, a new feature subset evaluation method is proposed by constructing a sample graph (SG) in different *k*-features and applying community modularity to select highly informative features as a group. However, these features may not be relevant as an individual. Furthermore, relevant in-dependency rather than irrelevant redundancy among the selected features is effectively measured with the community modularity Q value of the sample graph in the *k*-features. An efficient FS method called *k*-features sample graph feature selection is presented. A key property of this approach is that the discriminative cues of a feature subset with the maximum relevant in-dependency among features can be accurately determined. This community modularity-based method is then verified with the theory of k-means cluster. Compared with other state-of-the-art methods, the proposed approach is more effective, as verified by the results of several experiments.

Feature selection (FS) is widely investigated and utilized in machine learning and data mining research. In this context, a feature, also called attribute or variable, represents a property of a process or system. The goal of FS is to select the feature subsets of informative attributes or variables to build models that describe data and to eliminate redundant or irrelevant noise features to improve predictive accuracy^1^. FS not only maintains the original intrinsic properties of the selected features but also facilitates data visualization and understanding^2^. FS has been extensively applied to many applications, such as bio-informatics^3^, image retrieval^4^, and text classification^5^, because of its capabilities.

Traditional methods in FS can be broadly categorized into two approaches^6^, namely, *filter* and *wrapper* approaches. Filter algorithms^7^,^8^,^9^,^10^ utilize a simple weight score criterion to estimate the goodness of features. As a result, filter methods are classifier-independent and effective in terms of computational cost. However, filter methods disregard the correlations between features and provide feature subsets that may contain redundant information, which reduces classification accuracy. The correlation of concern in this study is a measure of the relationship between two mathematical variables (called features) or measured data values. In wrapper approaches^11^,^12^,^13^,^14^, feature subset selection depends on a classifier, which results in superior classification accuracy but requires high computational cost for repeated training of classifiers. Filter methods are eliciting an increasing amount of attention because of their efficiency and simplicity. This study focuses on filter methods only.

FS involves two major approaches: individual evaluation (univariate) and subset evaluation (multivariate). The former, which is also known as variable ranking, assesses an individual feature by using a scoring function for relevance. Subset evaluation produces candidate feature subsets through a certain search strategy. Each candidate subset is evaluated by a certain evaluation measure and compared with the previous best subset based on this measure. Individual evaluation only selects relevant features as an individual. However, a variable that is completely useless by itself can result in a significant performance improvement when combined with others^15^. Therefore, individual evaluation methods have been criticized for disregarding these features with strong discriminative power as a group but with weak power as an individual^16^. Furthermore, individual evaluation cannot eliminate redundant features because redundant features are likely to have similar rankings. Subset evaluation can handle feature redundancy with feature relevance^17^. The combination of several best individual features selected by individual evaluation methods does not generally lead to satisfactory classification results because the redundancy among selected features is not eliminated by individual evaluation methods^18^. Thus, subset evaluation method is considered the better approach between the two. Generally, the solution of a feature optimal subset is **NP**-hard^19^. To avoid the combinatorial search problem to find an optimal subset, variable selection methods are employed. The most popular of these methods mainly include *forward*^20^*, backward*^21^, and *floating sequential schemes*^22^, which adopt a heuristic search procedure to provide a sub-optimal solution.

In the subset evaluation method, evaluation of the relevance of a feature subset, including relevance and redundancy in a feature subset, is important in multivariate methods; however, this task is difficult in practice. Relevance evaluation methods based on mutual information (MI) have become popular recently^23^,^24^,^25^,^26^,^27^,^28^. However, these algorithms approximately estimate the discriminative power of a feature subset because loss of intrinsic information in raw data can occur while estimating the probability distribution of a feature vector by the discretization of a feature variable^27^,^28^.

A good feature subset should contain features that are highly correlated with the class but uncorrelated with one another^29^. In other words, in a good feature subset, the samples in different classes can be separated well; that is, the within-class distance in samples is small and between-classes distance is large. Therefore, if the samples are shown in a graph (also referred to as a complex network), the graph should exhibit obvious community structures^30^ and a high community modularity Q value^31^,^32^. Thus, the community modularity Q value can be utilized to evaluate the relevance of a feature subset with regard to the class. In this paper, a novel method is proposed to address the feature subset relevance evaluation problem by introducing a new evaluation criterion based on community modularity. The method accurately assesses the relevance independency of a feature subset by constructing a sample graph in different *k*-features. To the best of our knowledge, this work is the first to employ community modularity in feature subset relevance evaluation. The proposed method indiscriminately selects relevant features through the forward search strategy. This method not only selects relevant features as a group and eliminates redundant features but also attempts to retain intrinsic interdependent feature groups. The effectiveness of the method is validated through experiments on many publicly available datasets. Experimental results confirm that the proposed method exhibits improved FS and classification accuracy. The discriminative capacity of the selected feature subset is significantly superior to that of other methods.

## Related Work

FS has elicited increasing attention in the last few years. In the early stage, individual evaluation methods were more popular, such as those in^7^,^8^,^9^,^10^, which measure the discriminate ability of each feature according to a related evaluation criterion. Based on class information, these methods belong to the supervised FS algorithm. An unsupervised feature ranking algorithm has also been proposed; this algorithm considers not only the variance of each feature but also the locality preserving ability, such as the Laplacian score^33^.

A known limitation of individual evaluation methods is that the feature subset selected by these methods may contain redundancy^15^,^34^, which degrades the subsequent learning process. Thus, several subset evaluation-based filter methods, such as those in^17^,^29^,^35^,^36^,^37^, have been proposed to reduce redundancy during FS.

MI is gaining popularity because of its capability to provide an appropriate means of measuring the mutual dependence of two variables; it has been widely utilized to develop information theoretic-based FS criteria, such as *MIFS*^23^,^38^, CMIM^39^, CMIF^24^, MIFS-U^25^, *mrmr*^27^, NMIFS^28^, and FCBF^40^. MI is calculated with a Parzen window^41^, which is less computationally demanding and provides better estimation. The Parzen window method is a non-parametric method to estimate densities. It involves placing a kernel function on top of each sample and evaluating density as the sum of the kernels. The author in^42^ pointed out that common heuristics for information-based FS (including Markov Blanket algorithms^43^ as a special case) approximately and iteratively maximize the conditional likelihood. The author presented a unifying framework for information theoretic-based FS, bringing almost two decades of research on heuristic filter criteria under a single theoretical interpretation. Analysis of the redundancy among selected features is performed by computing the relevant redundancy between the features and the target. However, MI-based FS methods have been criticized for their limitations. First, loss of intrinsic information in raw data could occur because the probability distribution of the feature vector is estimated by the discretization of the feature variable. The second limitation is that these methods only select relevant features as an individual and disregard these informative features as a group^44^. Several researchers have also found that combining optimal features as an individual does not provide excellent classification performance^45^.

Graph-based methods, such as the Laplacian score^33^ and improved Laplacian score-based FS methods^46^,^47^,^48^,^49^, have been widely applied to feature learning because these approaches can evaluate the similarity among data. Generally, the graph-based method includes two phases. First, a graph is constructed in which each node corresponds to each feature, and each edge has a weight based on a criterion between features. Second, several clustering methods are implemented to select a highly coherent set of features^50^. Optimization-based FS algorithms are preferred by many researchers. R. Tibshirani^51^ proposed a new method called “lasso” for estimation in linear models. Based on graphical lasso (GL), a new multilink, single-task approach that combines GL with neural network (NN) was proposed to forecast traffic flow^52^.

Statistical methods have been widely applied to FS. Two popular feature ranking measures are *t*-test^53^ and *F*-statistics^54^. Well known statistic-based feature selection algorithms include *χ*^2^-statistic^55^, odds ratio^56^, bi-normal separation^57^, improved Gini index^58^, measure using Poisson distribution^59^, and ambiguity measure^60^. Most of these methods calculate a score based on the probability or frequency of each feature in bag-of-words to rank features according to a feature’s score; the top features are selected. Yan Wang^61^ introduced the concept of feature forest and proposed feature forest-based FS algorithm.

## Results

Experiments on artificial datasets, including binary class and multi-class datasets, were conducted to test the proposed approach. The proposed approach was also compared with several popular FS algorithms, including *MIFS_U*, *mrmr, CMIM*, Fisher, Laplacian score^33^, RELIEF^62^, Simba-sig^63^, and Greedy Feature Flip (G-Flip-sig)^63^. Off-the-shelf codes^42^ were used to implement *MIFS_U*, *mrmr*, and *CMIM* methods.

To evaluate the effectiveness of the proposed method, the nearest neighborhood classifier (1NN) with Euclidean distance and support vector machine (SVM)^64^ using the radial basis function and the penalty parameter *c* = 100 were employed to test the performance of the FS algorithms. We utilized the LIBSVM package^65^ for SVM classification. All experiments were conducted on a PC with Intel(R) Core(TM) i3-2310 CPU@2.10 GHz and 2G main memory.

### Datasets and preprocessing

To verify the effectiveness of the proposed method, six continuous datasets from the LIBSVM datasets^65^, two cancer microarray datasets, and two discrete datasets from UCI were utilized in the simulation experiments. All the features in the datasets, except discrete features, were uniformly scaled to zero mean and unit variance. The details of the 10 datasets are shown in Table 1.

### Feature selection and classification results

Classification performance was utilized to validate the FS method, and tenfold cross validation was employed to avoid the over-fitting problem. To reduce unintentional effects, all the experimental results are the average of 10 independent runs. In comparing the different methods, the feature subset was produced by picking the top ***s*** selected features to access each method in terms of classification accuracy (*s* = 1, ..., *P*). We discretized continuous features to nine discrete levels as performed in^66^,^67^ by converting the feature values between *μ* − *σ*/2 and *μ* + *σ*/2 to 0, the four intervals of size *σ* to the right of *μ* + *σ*/2 to discrete levels from 1 to 4, and the four intervals of size *σ* to the left of *μ* − *σ*/2 to discrete levels from −1 to −4. Extremely large positive or small negative feature values were truncated and discretized to ±4 appropriately.

**Figure 1:**
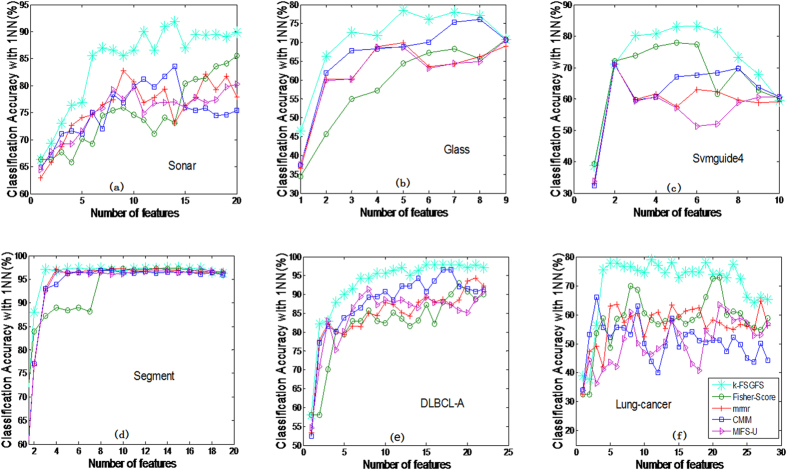
The average classification accuracy using 1NN classifier with respect to the subset of ***s*** features selected by different methods. For different methods, (**a**) is the classification accuracy with 1NN in data Sonar, (**b**) is the classification accuracy with 1NN in data Glass, (**c**) is the classification accuracy with 1NN in data Svmguide 4, (**d**). Is the classification accuracy with 1NN in data Segment, (**e**) is the classification accuracy with 1NN in data DLBCL-A, (**f**) is the classification accuracy with 1NN in data Lung-cancer.

**Figure 2:**
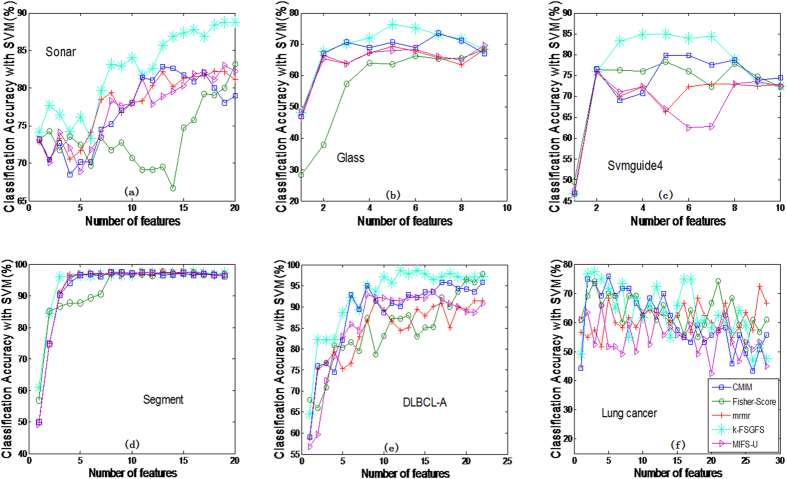
The average classification accuracy using SVM classifier with respect to the subset of ***s*** features selected by different methods. For different method, (**a**) is the classification accuracy with SVM classifier in data Sonar, (**b**) is the classification accuracy with SVM classifier in data Glass, (**c**) is the classification accuracy with SVM classifier in data Svmguide 4, (**d**) is the classification accuracy with SVM classifier in data Segment, (**e**) is the classification accuracy with SVM classifier in data DLBCL-A, (**f**) is the classification accuracy with SVM classifier in data Lung-cancer.

Table 2 indicates the average classification accuracy of both **1NN** and **SVM** classifiers at different ***s***. A bold value indicates the best among the FS methods under the same classifier and the same number of selected features. To avoid the influence of data scarcity, the average value of accuracy at different *s* for all datasets in the same selector is shown in the bottom line of Table 2 (*Avg*.). The results in Table 2 indicate that the proposed method (*k*-FSGFS) exhibits the best average performance compared with other methods in both classifiers. The *Avg*. values are 83.65% and 83.97% in 1NN and SVM classifiers, respectively. These values are higher than those of the other methods. CMIM is superior to *mrmr* and MIFS_U. Figures 1 and 2 show the performance of SVM and 1NN at different *s* of selected features for six datasets, namely, **Sonar, Glass, Svmguide4, Segment, DLBCL_A, and Lung-cancer.** The six datasets were selected because they cover a diverse range of characteristics, including continuous and discrete data, in terms of the number of features and number of examples.

Figures 1 and 2 show that the proposed method (*k-FSGFS*) outperforms the other methods. In most cases, the average accuracy of the two classifiers is significantly higher than that of other selectors. High classification accuracy is commonly achieved with minimal selected features, which indicates that our evaluation criterion based on community modularity Q not only selects the most informative features but also provides the solution of relevant independency among selected features. The proposed method can evaluate the discriminatory power of a feature subset.

**Figure 3:**
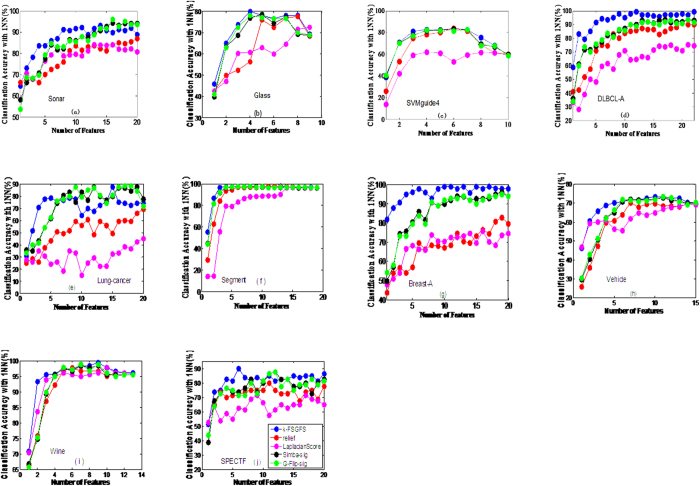
The average classification accuracy using 1NN classifier with respect to the subset of ***s*** features selected by other popular methods. (**a**) is the classification accuracy with 1NN classifier in data Sonar, (**b**) is the classification accuracy with 1NN classifier in data Glass, (**c**) is the classification accuracy with 1NN classifier in data Svmguide 4, (**d**) is the classification accuracy with 1NN classifier in data DLBCL-A, (**e**) is the classification accuracy with 1NN classifier in data Lung-cancer, (**f**) is the classification accuracy with 1NN classifier in data Segment, (**g**) is the classification accuracy with 1NN classifier in data Breast-A, (**h**) is the classification accuracy with 1NN classifier in data Vehicle, (**i**) is the classification accuracy with 1NN classifier in data Wine, (**j**) is the classification accuracy with 1NN classifier in data SPECTF.

Additionally, the proposed approach was compared with other popular FS methods, including Laplacian score^33^, Relief^62^, Simba-sig^63^, and Greedy Feature Flip (G-Flip-sig)^63^. Relief^62^, Simba-sig^63^, and G-Flip-sig^63^ are margin-based FS or feature weighting methods, in which a large nearest neighbor hypothesis margin ensures a large sample margin. Thus, these algorithms find a feature weight vector to minimize the upper bound of the leave-one-out cross-validation error of a nearest-neighbor classifier in the induced feature space. For fairness, only the 1NN classifier was utilized to evaluate the performance of the compared FS algorithms in all the datasets. Figure 3 shows that the proposed method is also superior or comparable to other methods in most cases. Particularly, the proposed method can achieve significantly higher classification accuracy in the first several features than the other methods in most cases. To verify, the classification accuracy results with the **1NN** classifier at different selected features *s* (*s* = 2, 3, 4) for different methods are illustrated in Table 3. The table clearly indicates that our method significantly improves the classification results with fewer selected features. Thus, our method achieves optimal performance with an acceptable number of features.

**Figure 4:**
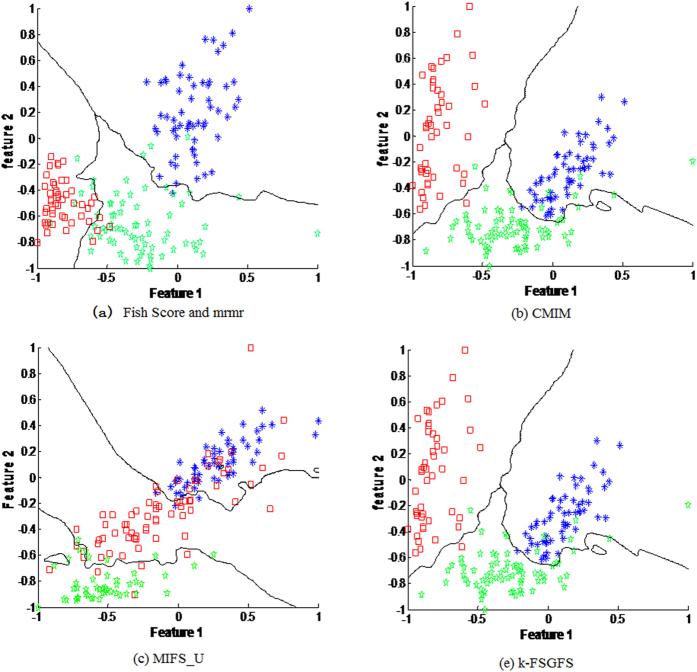
Decision boundary of 1NN classifier of samples with the two best informative features for different methods. Three colors represent three classes. (**a**) the decision boundary of 1NN classifier of samples in the two best informative features by Fish Score and mrmr. (**b**) the decision boundary of 1NN classifier of samples in the two best informative features by CMIM. (**c**) the decision boundary of 1NN classifier of samples in the two best informative features by MIFS_U. (**d**) the decision boundary of 1NN classifier of samples in the two best informative features by our method. From the results, both our method and CMIM have the lower classification error.

To further confirm the effectiveness of this feature evaluation criterion, the decision boundary of the 1NN classifier in 2D feature spaces from the **Wine** database was used, as shown in Fig. 4(a–d). The indicated dimensions are the two best features selected by each method. The two features selected by *k-FSGFS* and CMIM are relatively informative (Fig. 4(d)) and help in effectively separating the sample data. Both Fish Score and *mrmr* selected the same top two features, as indicated in Fig. 4(a), and separated the samples better than MIFS_U in Fig. 4(c). The proposed approach achieves high accuracy in classifying the samples in the two best informative feature spaces based on the results of the **Wine** dataset in Table 2.

The capability of *k-FSGFS* to obtain the discriminatory attribute of a feature subset and the relevant independency among features is so effective that it can select these informative features with fewer redundancies. Thus, *k-FSGFS* performs better than other FS algorithms. For parameter *K* during the construction of *k*-FSG in our method, numerous experiments demonstrate that a value of *K* selected from 2 to 11 is effective for most datasets for either SVM or 1NN classifier. In this study, *K* was set to 2.

### Statistical test

The classification experiments demonstrated that the proposed framework outperforms the other FS algorithms. However, the results also indicate that *k*-FSGFS does not perform better than several algorithms in a number of cases. Therefore, *paired sample one-tailed test* was used to assess the statistical significance of the difference in accuracy. In this test, the null hypothesis states that the average accuracy of *k*-FSGFS at different numbers of subsets is not greater than that of the other FS algorithms in terms of classification. Meanwhile, the alternative hypothesis states that *k*-FSGFS is superior to other FS algorithms in terms of classification. For example, if the performance of *k*-FSGFS is to be compared with that of *Fisher Score* method (k-FSGFS *vs*. Fish Score), the null and alternative hypotheses can be defined respectively as follows: H_0_: *μ*_*k*−*FSGFS*_ ≤ *μ*_*Fish*_*Score*_ and H_1_: *μ*_*k*−*FSGFS*_ > *μ*_*Fish*_*Score*_, where *μ*_*k*−*FSGFS*_ and *μ*_*Fish*_*Score*_ are the average classification accuracy of *k-FSGFS* and *Fish Score* methods at different numbers of selected features, respectively. The significance level was set to 5%. Tables 4 and 5 indicate that regardless of whether 1NN or SVM is used, the *p*-values obtained by the pair-wise one-tailed *t-test* are substantially less than 0.05, which means that the proposed *k*-FSGFS significantly outperforms the other algorithms.

### Justification of *k-FSGFS* based on K-means cluster

The justification of the proposed feature evaluation criterion based on community modularity was demonstrated by adopting the theory of K-means cluster to determine why *k* features with a higher Q value are more discriminative.

The K-means cluster^68^ is the most well-known clustering algorithm. It iteratively attempts to address the following objective: given a set of points in a Euclidean space and a positive integer *c* (the number of clusters), the points are split into *c* clusters to minimize the total sum of the Euclidean distances of each point to its nearest cluster center, which can be defined as follows:


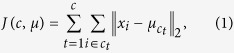


where *x*_*i*_ and 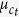 are the *i*-th sample point and its nearest cluster center, respectively, and 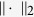 is the L_2_-norm.

In the feature weighting K-means, the feature that minimizes within-cluster distance and maximizes between-cluster distance is preferred, thus obtaining higher weight^56^. Confirming whether the features with a high community modularity Q value in our method can minimize within-cluster distance and maximize between-cluster distance is necessary.

According to Equation (7), 
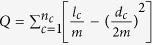
. Increasing the Q value equivalently maximizes inner edges *l*_*c*_ and minimizes outer edges *d*_*c*_, 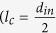, *d*_*c*_ = *d*_*out*_). In other words, each community of *k*-FSGs in *k*-features exhibits a large inner-degree *d*_*in*_(small out-degree *d*_*out*_), and the sample points in the *k*-features space with the same labels can be correctly classified as many as possible into the same class and as few as possible into different classes while these *k* features are good features as a group. The expected number of sample points in the *k*-features space that are correctly classified can be calculated through Neighborhood components analysis^69^.

Given the selected feature subset **S** and candidate features *f*, each sample point *i* in *S* ∪ *f* feature space selects another sample point *j* as its neighbor with probability *P*_*ij*_. *P*_*ij*_ can be defined by a soft max over Euclidean distances as follows:


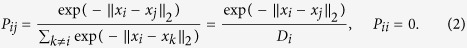


Under this stochastic selection rule, we can compute the probability *P*_*i*_ that point *i* will be correctly classified (denote the set of points in the same class as *i* by *C*_*t*_ = {* j*|*c*_*t*_ = *c*_*j*_}).


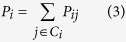


Hence, the expected number of sample points in the *S* ∪ *f* space correctly (*ENC*) classified into the same class is defined by


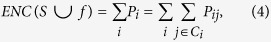



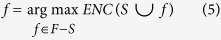


Feature *f* with larger *ENC* is more discriminative.

According to Eqs. 2, 3, 4, maximizing ENC is mutually equivalent to minimizing the K-means cluster objective *J*(*c*, *μ*).

(1) **Proof:** minimizing *J*(*c*, *μ*) ⇒ maximizing *ENC*(*f* ∪ *S*)

Given feature *f* ∈ *F* − *S*, Eq.2 is substituted into Eq.4. Thus, 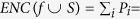 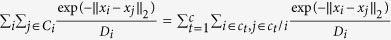

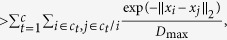
 *D*_max_ = max{*D*_1_, *D*_2_, ..., *D*_*n*_} 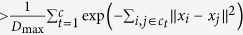 *c* is the number of clusters.

The lower bound of *ENC*(* f* ∪ *S*) is defined by *ENC*_*L*_*bound*._

*ENC*( *f* ∪ *S*) can be maximized simultaneously by maximizing its lower bound *ENC*_*L*_*bound*_ and equivalently minimizing 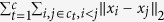.

As we know, 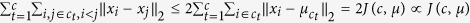, which denotes that lower bound *ENC*_*L*_*bound*_ has been maximized. *ENC*(*f* ∪ *S*) obtains the maximum value when the K-means objective (Eq. 1) is optimized for the minimum.

(2) **Proof:** maximizing *ENC*(*f*  ∪ *S*) ⇒ minimizing *J*(*c*, *μ*)

Based on the results in **proof** (1), 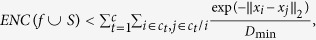 *D*_min_ = min{*D*_1_, *D*_2_, ..., *D*_*n*_} 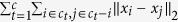 is equivalent to minimize while maximizing the *ENC*(*f* ∪ *S*), and because 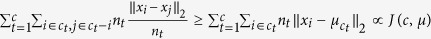 Hence, k-means cluster function *J*(*c*, *μ*) is minimized while 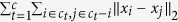 is minimized and *ENC*(*f* ∪ *S*) is maximized.

*J*(*c*, *μ*) in the *S* ∪ *f* space must be minimized when the community modularity Q value of SG in *S* ∪ *f* space obtains a high value, which indicates that the features selected by the proposed method can minimize within-cluster distance. Similarly, the expected number of points incorrectly classified is defined by *ENIC*(*f* ∪ *S*) = *n* − *ENC*(*f* ∪ *S*), where *n* is the number of samples. A small *ENIC*(*f* ∪ *S*) results in a few edges between communities and large between-cluster distance. The feature subset with a high Q value is highly relevant, which not only minimizes within-cluster distance but also maximizes between-cluster distance.

## Discussion

In this study, a novel feature subset evaluation criterion using the community modularity *Q* value by constructing *k*-features sample graphs (*k*-FSGs) is presented to measure the relevance of the feature subset with target variable C. To address the redundancy problem of ranking in filter methods, the sample graph in *k*-features that captures the relevant independency among feature subsets is utilized rather than the conditional MI criteria. By combining the two points above, a new FS method, namely, *k-FSGFS*, is developed for feature subset selection. The method effectively retains as many interdependent groups as possible during FS. The proposed *k-FSGFS* works well and outperforms other methods in most cases. The method remarkably or comparatively improves FS and classification accuracy with a small feature subset, which demonstrates the ability of the proposed method to select a discriminative feature subset. The experimental results also verify that interdependent groups commonly exist in the real dataset and play an important role in classification. Unlike the other methods used for comparison, the proposed method accurately evaluates the discriminative power of a feature subset as a group. The Fisher method, which is an individual evaluation criterion, cannot eliminate the redundancy in a feature subset, thereby reducing classification performance. The experiment results for the Fisher method verify this finding. The MI-based methods, such as *mrmr*, MIFS_U, and CMIM, consider the relevance and redundancy among feature subsets as a group and are superior to the Fisher method. However, these MI-based methods can only approximately estimate the relevance and redundancy in a feature subset (such as considering all the redundancy between pair-wise features to estimate the redundancy among a feature subset as a group in *mrmr* method) because of the difficulties in accurately computing the probability density function. The results in Table 2 and Figs 1, 2 indicate that *mrmr*, MIFS_U, and CMIM methods perform better than the Fisher method but worse than the proposed method.

From the mentioned above, our method perform better than MI-based methods in most cases. In our method, larger inter-class distance implies that the local margin of any sample should be large enough. By the large margin theory^70^, the upper bound of the leave-one-out cross-validation error of a nearest-neighbor classifier in the feature space is minimized and usually generalizes well on unseen test data^70^,^71^. However, traditional mutual information based relevance evaluation between feature and class can not accurately measure the discriminative power of a feature. In order to better illustrate this, for simplicity, the features ***f***_***1***_, ***f***_***2***_and the class vector **C** are defined by as following:


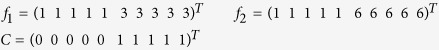


According to MI-based methods, the feature *f*_1_ has the same relevancy as *f*_2._ In our method, the feature *f*_2_ has more discriminative power than *f*_1_ because the community modularity *Q* in feature *f*_2_ is larger than feature *f*_1_. Intuitively, feature *f*_2_ should be more relevant than *f*_1_ due to its between-class distance is larger than *f*_1_. However, the MI-based method can not capture the difference between *f*_1_ and *f*_2._ Therefore, our relevancy evaluation criterion based on community modularity Q is more efficient and accurate.

**Figure 5:**
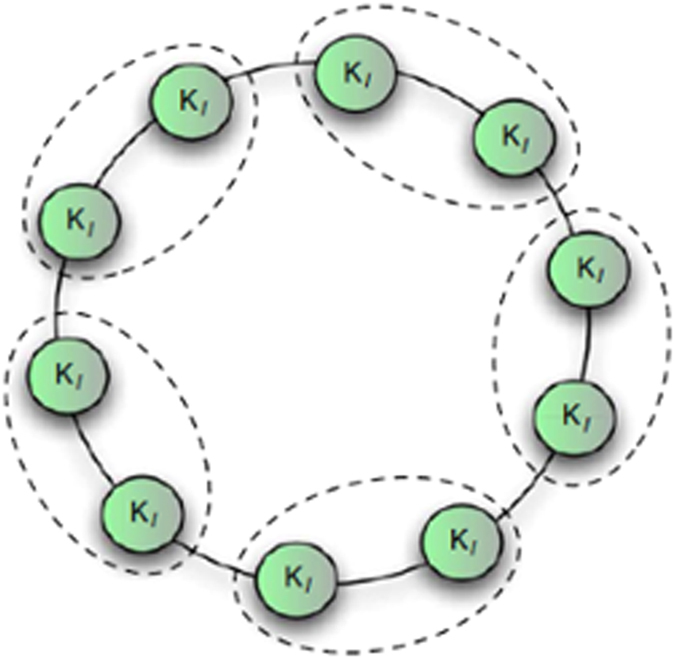
Resolution limit of modularity optimization. The natural community structure of the graph, represented by the individual cliques (circles), is not recognized by optimizing modularity, if the cliques are smaller than a scale depending on the size of the graph. In this case, the maximum modularity corresponds to a partition whose clusters include two or more cliques (like the groups indicated by the dashed contours)^72^.

However, in practice, the proposed method is not always efficient for all types of datasets, such as imbalanced datasets, especially when a few samples in one class are compared with other classes. For example, in the dataset Lung-cancer, our method performs worse than simba-sig and G-flip-sig. Because, modularity optimization is widely criticized for its resolution limit^72^ illustrated in Fig. 5, which may prevent the approach from detecting clusters. The clusters are comparatively small with respect to the graph as a whole, which results in maximum modularity Q not corresponding to a good community structure, that is, features with a high Q value may be irrelevant. The KNN searching needs to be conducted iteratively in our method, thus, the efficiency of our method is low for larger data amounts in real applications with regard to time complexity. Our future work will focus on resolving these problems.

## Methods

In this paper, a new feature evaluation criterion based on the community modularity Q value is proposed to evaluate the class-dependent correlation^73^ of features as a group instead of identifying the discriminatory power of a single feature. Detailed information on our method is presented in Algorithm 2. The innovations of our work mainly include the following points.

1. The discriminatory power of features as a group can be evaluated exactly based on the community modularity Q value of sample graphs in *k*-features.
2. The proposed method can select features that have discriminatory power as a group but have weak power as an individual.
3. Relevant independency instead of irrelevant redundancy between features is measured using the community modularity Q value rather than information theory.

**Figure 6:**
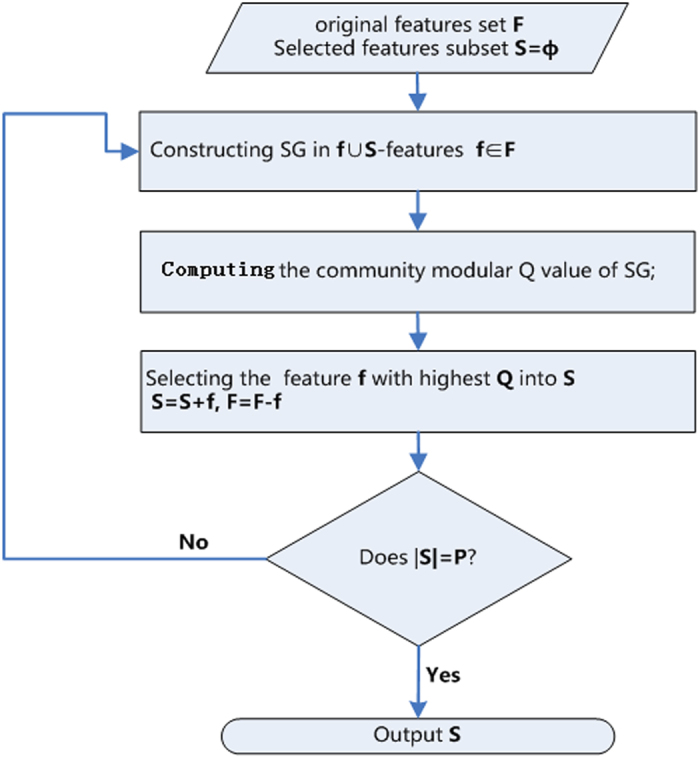
The flow diagram of proposed method (*k*-FSGFS), mainly including three steps: i) constructing the SGs in *f* ∪ *S* features (*f* ∈ *F*), ii) computing the community modular Q value of SGs, iii) selecting the feature ***f*** with the largest Q value into the selected features subset **S**. The iterative process terminates until the |*S*| = *P*.

The proposed framework is presented in a flow diagram in Fig. 6.

### Community modularity Q

**Figure 7:**
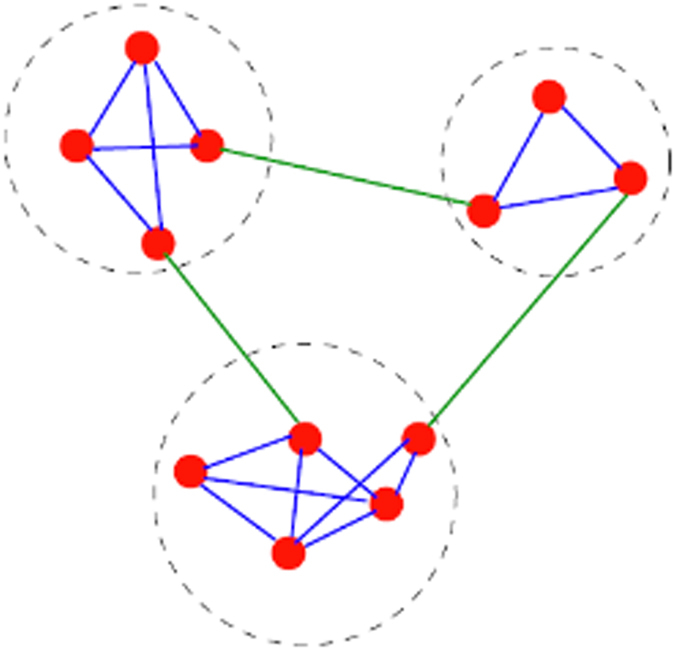
A simple graph with three communities, enclosed by the dashed circles. Reprinted figure with permission from^72^.

The community structure in an undirected graph exhibits close connections within the community but sparse connections among various communities relatively^31^,^32^. Figure 7 shows a schematic example of a graph with three communities to demonstrate the community structure.

Thus far, the most regarded quality function is the modularity of Newman and Girvan^32^. Modularity *Q* can be written as follows:


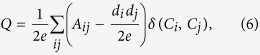


where the sum runs over all pairs of nodes, *A* is the adjacency matrix, *e* is the total number of edges of the graph, and *d*_*i*_ and *d*_*j*_ represent the degree of nodes *i* and *j*, respectively. The *δ*-function is equal to one if nodes *i* and *j* are in the same community and equal to zero otherwise. Another popular description of modularity *Q* can be written as follows:


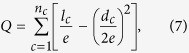


where *n*_*c*_ is the number of communities, *l*_*c*_ is the total number of edges joining the nodes of module *C*, and *d*_*c*_ is the sum of the degrees of the nodes of *C*. The range of modularity *Q* is [−1, 1]. Modularity-based methods^23^ assume that a high value of modularity indicates good partitions. In other words, the higher modularity *Q* is, the more significant the community structure is.

Based on the definition of community, the within-class distance in a community is small and the between-class distance is large. Thus, if a graph has a clear community structure, the nodes in different communities can be locally and linearly separated easily, as shown in Fig. 7. The features that minimize within-cluster distance and maximize between-cluster distance are preferred and obtain a high weight. If the sample graph in *k*-features (*k*-FSG) has an apparent community structure, these *k* features will have strong discriminative power as a group because intra-class distance is small and inter-class distance large. This condition can be proven sequentially with the theory of K-means cluster.

### Sample graph in *k*-features (*k*-FSG)

Given an *m* × *n* dataset matrix (*m* corresponding to samples and *n* corresponding to features), the sample graph in *k*-features (*k*-FSG) can be constructed as follows: an edge *A*(*i*, *j*) (*A*(*i*, *j*) = 1) exists between samples *X*_*i*_ and *X*_*j*_ if *X*_*i*_ ∈ *K* − *NN*(*X*_*j*_) or *X*_*j*_ ∈ *K* − *NN*(*X*_*i*_).where *X*_*i*_ is the node *i* corresponding to the sample *i*, *K* − *NN*(*X*_*i*_) is the *K*-neighborhood set of node *i*, and **A** is the adjacency matrix, which is symmetrical. *K* is the predefined parameter and does not have large values, which generally range within {3–11}.

The discussion above indicates that if *k*-FSG in *k*-features exhibits clear community structures corresponding to a large Q value, these *k* features are highly informative as a group. The algorithm of constructing *k*-FSG is shown as **Algorithm 1.**

**Algorithm 1:** Pseudo-code for constructing *k*-FSG


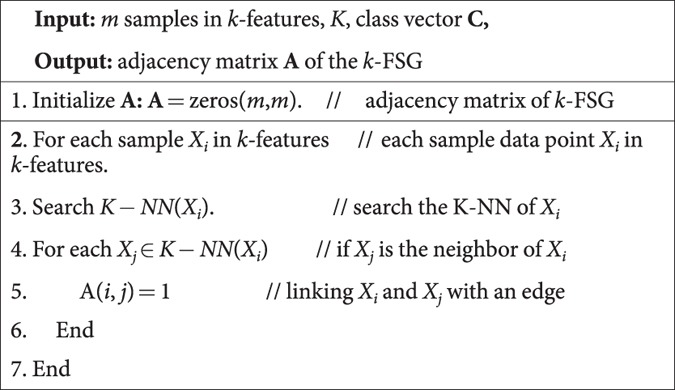


### Feature subset selection with sample graph in *k*-features

**Figure 8:**
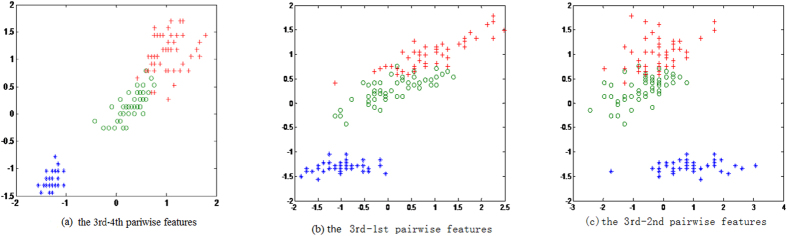
The sample scatter points in 2-F*SGs* (*k* = 2) of three pairs features in *iris* dataset. The different color corresponds to different classes. (**a**) The sample scatter points in features 3 and 4. (**b**) The sample scatter points in features 3 and 1. (**c**) The sample scatter points in features 3 and 2. From the sample scatter points results, it can be concluded that the sample points in features 3 and 4 can be easily separated, which means the features 3 and 4 as a group have more discriminative power.

In this subsection, a novel *k*-FSG-based feature selection method (*k*-FSGFS) for ranking features is proposed based on *k*-FSG and community modularity Q. First, all the sample graphs in 1D feature space (*k* = 1) can be constructed based on **Algorithm 1**. The most informative feature is ***f***_***1***_, where the sample graph in ***f***_***1***_(1-feature) enables the largest community modularity Q value to be selected. Given feature ***f***_***1,***_all the sample graphs in a two-feature space (*k* = 2) (*f*_1_ *and q* ∈ *F* − *f*_1_ space) and all the community modularity 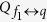 values of the two FSGs are calculated. Feature ***q*** with the highest 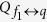 values will be selected in feature subset **S**. The procedure will not stop until the number of selected features satisfies **|S|** = P. To facilitate understanding of our evaluation scheme, we regard a UCI dataset, *iris*, as an example. The dataset consists of 150 samples and four features. The dataset is divided into three classes with 50 samples in each class. The *iris* dataset is processed with zero mean and unit variance according to 1-FSG in one feature. The 3rd feature with the highest Q value is the most informative as an individual. Given the 3rd feature, Fig. 8 illustrates the sample scatter points in 2-FSG*s* for the remaining features {1 2 4} in dataset *iris*. Three community modularity *Q*_3↔*q*_ values are shown in Table 6 (q = 1, 2, 4). Figure 8 clearly indicate that the 2-FSG in 3 ↔ 4 feature space exhibits more obvious community structures, and the sample points in different classes in 3 ↔ 4 features can be easily separated. The results in Table 6 show that the 2-FSG in 3 ↔ 4 feature space provides the largest community modularity Q value. Thus, the 4th feature has strong informative power combined with the 3rd feature. Given the 3rd and the 4th features, the 1st and the 2nd features can be selected according to the 3-FSGs and 4-FSGs, respectively. The selected feature subset in *iris* using our method is {3 4 1 2}, which is the selected features of most of the methods.

In short, given selected feature subset **S**, feature ***f*** selected by our criterion can be defined as follows:


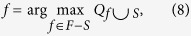


where *Q*_*f*∪*S*_ is the community modularity value of SG in features *f* ∪ *S* and *F* and *S* are the set of all features and selected feature subset, respectively.

### Relevancy analysis

**Figure 9:**
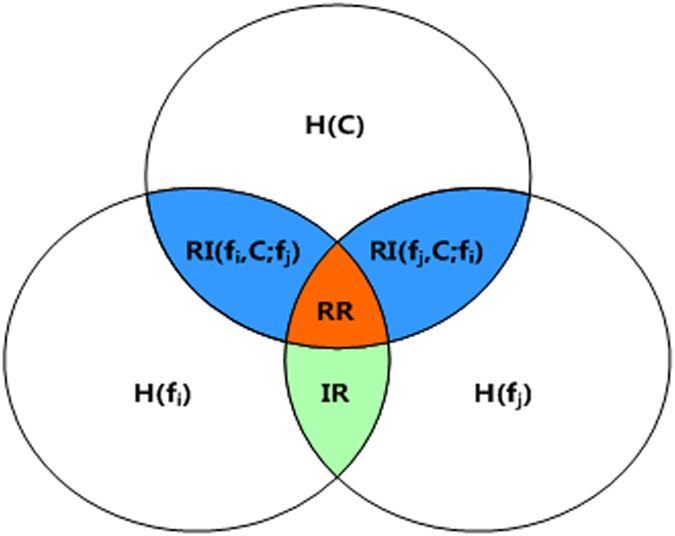
**V**isualization of *IR, RR* and *RI* between features *f*_*i*_ and *f*_*j*_, where *H*(*f*_*i*_) (*H*(*f*_*j*_)) is the entropy of feature *f*_*i*_ (*f*_*j*_), and *H*(*C*) is the entropy of class variable. The shaded area IR is the class-independent correlation between features *f*_*i*_ and *f*_*j*_ the shaded area is the class-dependent correlation between features *f*_*i*_ and *f*_*j*_ with respect to classy. The shaded area RI to what we refer as relevant independency, that is, the amount of information two random variables can predict about a relevant one and it is not shared by each other. See text for details.

Ranking-based filter methods cannot handle high redundancy among the selected features. To solve this problem, conditional MI (CMI) is applied in this study to obtain the relevant independency (RI) or relevant redundancy^74^ instead of the irrelevant redundancy between features, as shown in Fig. 9. *RI*(*f*_*i*_, *C*; *f*_*j*_) is now the amount of information features *f*_*i*_ that can predict target variable *C* when feature *f*_*j*_ is given; *RI*(*f*_*i*_, *C*; *f*_*j*_) = *I*(*f*_*i*_, *C* | *f*_*j*_). Similarly, *RI*(*f*_*j*_, *C*; *f*_*i*_). In other words, if *RI*(*f*_*i*_, *C*; *f*_*j*_) between features *f*_*i*_ and *f*_*j*_ is large, the combination of feature *f*_*i*_ can provide informative information when feature *f*_*j*_ is selected. However, calculating *RI*(*f*_*i*_, *C*; *S*) when selected feature subset S is given is difficult for MI-based methods. The first reason is that examples are often insufficient. Second, accurate estimation for multivariate density *P*(*f*_1_, *f*_2_, ..., *f*_*n*_, *C*) and *P*(*f*_1_, *f*_2_, ..., *f*_*n*_) is difficult. For the MI-based methods, such as *MIFS, mrmr, MIFS_U, CMIM, and CMIF*, *RI*(*f*_*i*_, *C*; *f*_*j*_) are often approximated in different ways. Therefore, MI-based methods cannot exactly evaluate *RI*(*f*_*i*_, *C*; *S*).

In this study, the discriminative capability of *k* features as a group was evaluated using the community modularity Q value of the constructed *k*-FSG. A high Q value of *k*-FSG denotes large RI among the *k* features as a group, and the sample points in different classes can be separated well. Thus, the community modularity Q value of *k*-FSG in *k*-features can accurately illustrate relevant independency *RI*(*f*_*i*_, *C*; *S*) in selected feature subset S. The community modularity Q value of *k*-FSG was utilized to measure relevant independency instead of MI theory. For verification, the *iris* dataset was used as an example. Different *RI*(*f*_*i*_, *C*; *f*_3_) values were calculated, and the third feature was selected (*i* = 1, 2, 4), as indicated in Table 7 The table clearly indicates that *RI*(*f*_4_, *C*; *f*_3_) is the largest, which demonstrates that fourth feature *f*_4_ can provide more informative information when the third feature is given. Similarly, the *Q*_3↔4_ value in Table 6 is also the highest in Table 7, which demonstrates that the community modular Q value of *k*-FSG in *k*-features can replace MI to effectively evaluate the RI of feature subset **S.** Thus, our method can resolve relevant redundancy among selected features. CMI can be computed with the FEAST tool^42^.

Relevant independency *RI*(*f*_*i*_, *C*; *S*) between feature *f*_*i*_ and selected feature set *S* was replaced by the community modularity Q value of SG in *f*_*i*_ ∪ *S*, which can be defined as follows:


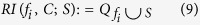


A larger value of *RI*(*f*_*i*_, *C*; *S*) indicates that *f*_*i*_ is highly independent with features in **S** but relevant in terms of target variable *C* and has strong informative power combined with features in **S**. These results indicate that our method can select these features with more relevancy as a group in terms of class and larger RI among selected features.

The details of *k-FSGFS* are presented in **Algorithm 2**.

**Algorithm 2:** *k*-FSGFS: *k*-features sample graph based feature selection


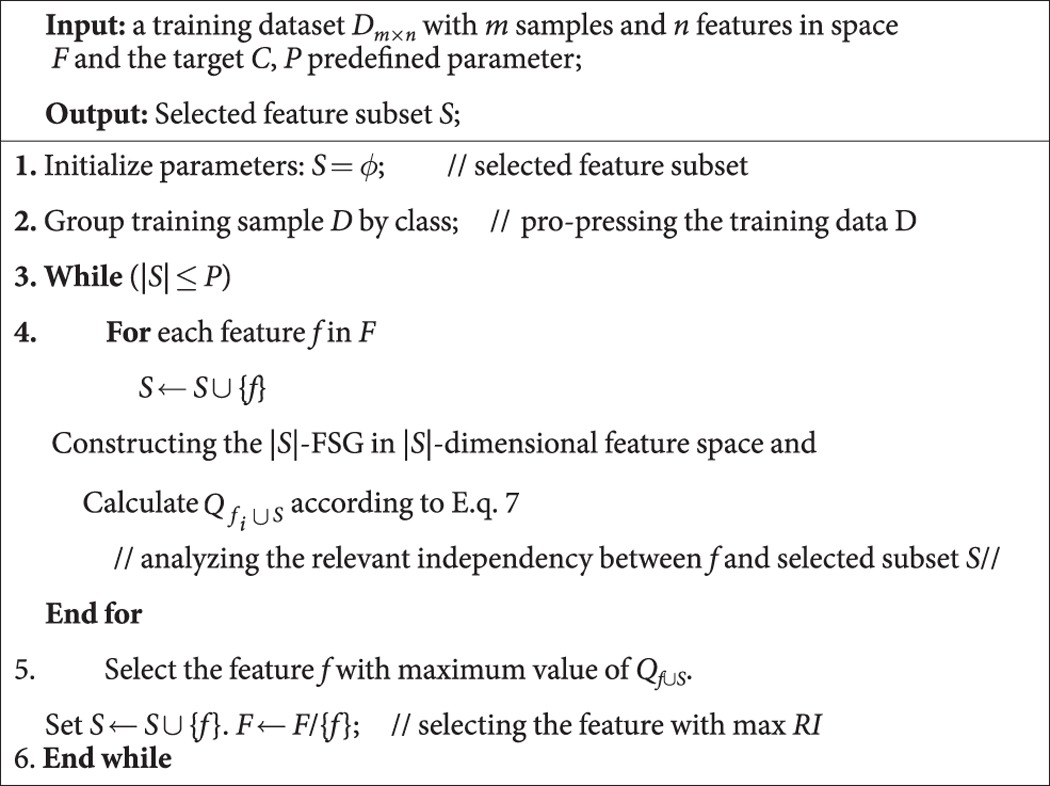


### Time complexity of *k-FSGFS*

Algorithm 2 shows that *k*-FSGFS mainly includes two steps. The first step is to construct *k-FSG* in *k*-features space. The second step is to calculate the community modularity *Q* value of each *k-FSG*. The most time-consuming step is establishing *k*-FSG, whose time complexity is about *ο*(*Pnm*^2^), where *n* is the number of features in feature space, *m* is the number of samples in the dataset, and P is the number of predefined selected features. Fortunately, fast K-nearest neighbor graph construction methods^75^,^76^ can be applied to the construction of *k*-FSGs; such application would reduce the time complexity from *ο*(*Pnm*^2^) to *ο*(*Pnm*^1.14^). In the second step, the spending time is approximately *ο*(*m*log *m*). Thus, the overall time cost of *k*-FSGFS is approximately 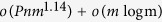.

## Additional Information

**How to cite this article**: Zhao, G. and Liu, S. Estimation of Discriminative Feature Subset Using Community Modularity. *Sci. Rep*. **6**, 25040; doi: 10.1038/srep25040 (2016).

## Figures and Tables

**Table 1:** Characteristics of the data sets in our experiment.

No.	Dataset	Sample	Features	Classes	Source	style
1	Wine	178	13	3	Libsvm dataset	continuous
2	Sonar	208	60	2	Libsvm dataset	continuous
3	Svmguide4	300	10	6	Libsvm dataset	continuous
4	Glass	214	9	6	Libsvm dataset	continuous
5	Vehicle	846	18	4	Libsvm dataset	continuous
6	Segment	2310	19	7	Libsvm dataset	continuous
7	DLBCL-A	141	661	3	77	continuous
8	Breast-A	98	1213	3	77	continuous
9	Lung-cancer	32	56	3	UCI	discrete
10	SPECTF	80	44	2	UCI	discrete

**Table 2:** Average accuracy (%) for different FS algorithms based on 1NN and SVM classifier, respectively.

1NN accuracy						SVM accuracy					
#R	*Fisher*	*CMIM*	*MIFS_U*	*mrmr*	*k-FSGFS*	#R	*Fisher*	*CMIM*	*MIFS_U*	*mrmr*	*k-FSGFS*
Wine (n = 13)											
s = 2	84.41	92.84	78.08	85.58	92.84	s = 2	90.59	94.55	82.58	91.59	**94.55**
s = 5	95.32	94.57	94.56	94.91	**97.02**	s = 4	90.82	95.12	92.7	92.67	**95.89**
s = 7	96.07	97.57	94.21	97.38	**98.31**	s = 6	99.25	95.67	95.32	99.25	**99.43**
Sonar (n = 60)											
s = 10	74.57	79.85	79.78	80.73	**86.57**	s = 10	70.66	78.03	77.86	78.03	**83.96**
s = 15	80.35	76.03	75.95	76.38	**87.04**	s = 15	74.66	81.71	80.31	81.28	**87.33**
s = 20	85.54	75.45	80.33	77.95	**89.85**	s = 20	83.64	78.98	82.25	81.14	**88.73**
Glass (n = 9)											
s = 4	57.16	68.21	68.7	68.89	**71.86**	s = 4	63.87	68.78	67.17	67.11	**71.77**
s = 5	64.39	68.8	68.66	69.93	**78.49**	s = 5	63.51	70.39	67.78	69.32	**76.21**
s = 6	67.2	70.03	63.06	63.59	**76.14**	s = 6	66.06	68.83	68.23	67.71	**75.11**
Vehicle (n = 18)											
s = 2	56.26	48.93	49.2	48.02	**61.14**	s = 2	56.84	64.65	56.99	46.34	**65.85**
s = 4	60.05	64.93	58.83	51.71	**69.86**	s = 4	55.08	67.36	63.37	53.89	**74.47**
s = 6	64.31	68.99	61.82	61.27	**72.22**	s = 6	66.54	75.06	68.31	71.51	74.45
Svmguide4 (n = 10)											
s = 3	73.88	59.66	59.22	59.88	**80.11**	s = 3	76.11	68.89	71.04	69.87	**83.22**
s = 5	77.88	67.11	57.11	57.44	**83.02**	s = 5	78.11	79.78	66.78	66.11	**84.89**
s = 7	61.66	68.33	52.01	62.33	**81.22**	s = 7	72.13	77.44	62.78	73.02	**84.33**
Segment (n = 19)											
s = 1	**62.03**	45.36	44.89	45.1	56.32	s = 1	56.97	49.91	49.48	49.78	**61.04**
s = 2	83.93	77.05	76.96	76.62	**87.96**	s = 2	**84.98**	74.85	74.72	75.02	84.55
s = 3	87.22	93.03	92.55	93.11	**97.05**	s = 3	86.62	90.39	90.48	90.82	**96.11**
DLBCL-A (n = 661)											
s = 5	79.42	83.85	80.19	79.47	**90.09**	s = 5	80.24	82.19	82.9	75.29	88.62
s = 10	82.33	90.71	88.61	87.9	**95.71**	s = 10	83.05	88.67	92.14	89.33	**97.19**
s = 15	87.23	90.71	89.33	89.33	**97.85**	s = 15	85.05	93.62	92.14	87.9	**97.86**
Breast-A (n = 1213)											
s = 5	83.29	88.96	86.14	84.77	**94.55**	s = 3	80.66	82.66	80.66	81.66	**91.66**
s = 10	80.03	92.25	91.07	89.14	**94.25**	s = 5	74.55	87.88	78.88	82.77	**93.88**
s = 15	85.22	91.48	90.81	87.7	**97.96**	s = 7	75.55	86.77	91.03	79.44	**96.03**
Lung-cancer (n = 56)											
s = 8	70.02	53.33	60.83	59.44	**76.66**	s = 3	74.16	73.33	52.5	57.5	**77.5**
s = 12	56.66	40.04	48.33	60.83	**76.94**	s = 12	60.83	64.16	62.5	63.33	**72.5**
s = 16	56.94	53.33	48.61	61.38	**74.72**	s = 16	55.83	55.03	55.01	66.66	**75**
SPECTF (n = 44)											
s = 4	76.25	60.03	**80.02**	72.5	76.25	s = 2	57.91	60.41	58.33	55.83	**73.75**
s = 6	78.75	66.25	73.75	76.25	**82.5**	s = 5	80.41	67.5	71.25	72.08	**85.41**
s = 8	71.25	73.75	72.5	67.5	**85**	s = 9	72.91	66.66	66.25	67.08	**87.91**
***Avg.***	***74.65***	***73.37***	***72.21***	***72.9***	***83.65***		***73.92***	***76.28***	***73.78***	***73.44***	***83.97***

**Table 3:** Average accuracy (%) for other different FS algorithms based on 1NN classifier.

1NN accuracy					
#R	Relief	LaplacianScore	Simba-sig	G-Flip-sig	*k-FSGFS*
Wine (n = 13)					
s = 2	75.81	83.66	74.73	75.22	**93.26**
s = 3	86.99	93.88	89.28	89.80	**95.52**
s = 4	92.18	94.93	95.49	94.90	**95.55**
Sonar (n = 60)					
s = 2	67.74	70.76	66.33	68.71	**73.17**
s = 3	68.21	67.86	68.29	68.33	**78.31**
s = 4	66.36	70.17	70.76	69.81	**83.71**
Glass (n = 9)					
s = 2	49.89	47.12	63.18	62.97	**65.05**
s = 3	52.27	60.24	68.68	72.42	**73.68**
s = 4	56.21	60.78	76.69	78.03	**79.87**
Vehicle (n = 18)					
s = 2	35.81	59.32	40.42	42.89	**60.75**
s = 3	47.38	60.06	50.83	51.42	**65.85**
s = 4	59.79	60.87	62.28	61.94	**68.92**
Svmguide4 (n = 10)					
s = 2	53.33	42.33	70.67	70.33	**71.04**
s = 3	74.67	59.03	77.67	77.08	**81.07**
s = 4	78.33	62.04	81.67	82.33	**82.67**
Segment (n = 19)					
s = 2	62.68	14.51	81.65	81.52	**86.84**
s = 3	84.33	55.63	91.95	92.16	**96.58**
s = 4	93.38	79.52	96.75	96.84	**97.23**
DLBCL-A (n = 661)					
s = 2	42.33	28.33	42.33	60.24	**83.05**
s = 3	51.86	39.03	51.86	73.76	**79.43**
s = 4	57.52	50.29	57.52	70.19	**85.11**
Breast-A (n = 1213)					
s = 2	53.89	51.04	57.22	58.11	**87.89**
s = 3	56.78	54.11	74.33	73.33	**90.78**
s = 4	54.02	66.56	75.78	73.33	**95.06**
Lung-cancer (n = 56)					
s = 2	28.33	25.83	35.03	40.03	**51.67**
s = 3	25.83	35.06	43.33	41.67	**70.83**
s = 4	37.52	30.83	54.17	54.17	**77.51**
SPECTF (n = 44)					
s = 2	65.03	63.75	67.51	63.75	**73.75**
s = 3	73.75	53.75	72.51	72.51	**75.03**
s = 4	70.06	58.75	76.25	76.25	**82.51**
***Avg.***	***60.73***	***56.66***	***67.83***	***69.80***	***80.05***

**Table 4:** The *paired sample one-tailed test* results of *k-FSGFS* and other algorithms in 1NN and SVM.

Pair-wise t-test	1NN_*p*-value	SVM_*p*-value
*k*-FSGFS vs. Fish Score	1.51E-09	5.08E-11
*k*-FSGFS vs. CMIM	4.75E-08	5.91E-09
*k*-FSGFS vs. MIFS_U	4.75E-08	3.63E-10
*k*-FSGFS vs. mrmr	3.07E-11	4.28E-11

**Table 5:** The *paired sample one-tailed test* results of *k-FSGFS* and other algorithms in 1NN.

Pair-wise t-test	1NN_*p*-value
*k*-FSGFS vs. Relief	5.80E-10
*k*-FSGFS vs. LaplacianScore	6.28E-09
*k*-FSGFS vs. Simba-sig	2.53E-07
*k*-FSGFS vs. G-Flip-sig	1.53E-07

**Table 6:** The community modularity *Q* values of 2-FSG (*k* = 2) in different pairwise features in *iris* dataset.

2 − *FSG*_3↔*q*_	3 ↔ 4	3 ↔ 1	3 ↔ 2
*Q*_3↔*q*_	0.6057	0.5719	0.5430

The more larger the community modularity is, the more relevant the pairwise features are. The features 3 and 4 as a group have more discriminative power.

**Table 7:** The RI in different pairwise features in terms of the third feature in *iris* dataset.

*RI*	*RI* (*f*_1_, *C*; *f*_3_)	*RI* (*f*_2_, *C*; *f*_3_)	*RI* (*f*_4_, *C*; *f*_3_)
*value*	0.0221	0.0031	0.1358

The larger RI states that the features 3 and 4 as a group have more discriminative power.
